# SARS-CoV-2 Dissemination Through Peripheral Nerves Explains Multiple Organ Injury

**DOI:** 10.3389/fncel.2020.00229

**Published:** 2020-08-05

**Authors:** Matija Fenrich, Stefan Mrdenovic, Marta Balog, Svetlana Tomic, Milorad Zjalic, Alen Roncevic, Dario Mandic, Zeljko Debeljak, Marija Heffer

**Affiliations:** ^1^Laboratory of Neurobiology, Department of Medical Biology and Genetics, Faculty of Medicine Osijek, Josip Juraj Strossmayer University of Osijek, Osijek, Croatia; ^2^Department of Hematology, Clinic of Internal Medicine, University Hospital Osijek, Osijek, Croatia; ^3^Department of Internal Medicine, Family Medicine and History of Medicine, Faculty of Medicine Osijek, Josip Juraj Strossmayer University of Osijek, Osijek, Croatia; ^4^Clinic of Neurology, University Hospital Osijek, Osijek, Croatia; ^5^Department of Neurology and Neurosurgery, Faculty of Medicine Osijek, Josip Juraj Strossmayer University of Osijek, Osijek, Croatia; ^6^Department of Medical Chemistry, Biochemistry and Clinical Chemistry, Faculty of Medicine Osijek, Josip Juraj Strossmayer University of Osijek, Osijek, Croatia; ^7^Clinical Institute of Laboratory Diagnostics, University Hospital Osijek, Osijek, Croatia; ^8^Department of Pharmacology, Faculty of Medicine Osijek, Josip Juraj Strossmayer University of Osijek, Osijek, Croatia

**Keywords:** SARS-CoV-2, neurotropic infection, axonal transport, peripheral nerves, neurological symptoms, multiple organ failure

## Abstract

Coronavirus disease (CoVID-19), caused by recently identified severe acute respiratory distress syndrome coronavirus 2 (SARS-CoV-2), is characterized by inconsistent clinical presentations. While many infected individuals remain asymptomatic or show mild respiratory symptoms, others develop severe pneumonia or even respiratory distress syndrome. SARS-CoV-2 is reported to be able to infect the lungs, the intestines, blood vessels, the bile ducts, the conjunctiva, macrophages, T lymphocytes, the heart, liver, kidneys, and brain. More than a third of cases displayed neurological involvement, and many severely ill patients developed multiple organ infection and injury. However, less than 1% of patients had a detectable level of SARS-CoV-2 in the blood, raising a question of how the virus spreads throughout the body. We propose that nerve terminals in the orofacial mucosa, eyes, and olfactory neuroepithelium act as entry points for the brain invasion, allowing SARS-CoV-2 to infect the brainstem. By exploiting the subcellular membrane compartments of infected cells, a feature common to all coronaviruses, SARS-CoV-2 is capable to disseminate from the brain to periphery via vesicular axonal transport and passive diffusion through axonal endoplasmic reticula, causing multiple organ injury independently of an underlying respiratory infection. The proposed model clarifies a wide range of clinically observed phenomena in CoVID-19 patients, such as neurological symptoms unassociated with lung pathology, protracted presence of the virus in samples obtained from recovered patients, exaggerated immune response, and multiple organ failure in severe cases with variable course and dynamics of the disease. We believe that this model can provide novel insights into CoVID-19 and its long-term sequelae, and establish a framework for further research.

## Introduction

The ongoing pandemic of coronavirus disease (CoVID-19) has profoundly affected many aspects of our lives. The disease is caused by severe acute respiratory distress syndrome coronavirus 2 (SARS-CoV-2), a positive-sense single-stranded RNA beta-coronavirus that uses angiotensin converting enzyme 2 (ACE2) to invade host cells (Hoffmann et al., 2020a). CoVID-19 exhibits variable clinical presentations, ranging from mild respiratory and/or gastrointestinal symptoms to acute respiratory distress syndrome and multiple organ failure (Hani et al., 2020; Jiang et al., 2020; Lai et al., 2020; Pan et al., 2020). A significant number of apparently asymptomatic individuals were also reported (Day, 2020).

So far, SARS-CoV-2 was has been shown to infect bronchial, alveolar, and conjunctival epithelia, alveolar macrophages (Bao et al., 2020; Hui et al., 2020), T-lymphocytes (Wang et al., 2020c), neurons (Moriguchi et al., 2020; Paniz-Mondolfi et al., 2020), cholangiocytes (Zhao et al., 2020a), vascular endothelium (Varga et al., 2020), gastrointestinal mucosa (Xiao et al., 2020), the heart, liver, and kidneys (Puelles et al., 2020). It has been suggested that brain involvement might contribute to more complicated clinical presentations (Li et al., 2020; Steardo et al., 2020). According to initial reports, more than a third of hospitalized patients exhibited symptoms and signs of neuronal involvement (Mao et al., 2020), and speculations on neuroinvasive potential of the virus were promptly made (Toljan, 2020). We would like to propose that SARS-CoV-2, after infecting the targeted brain nuclei, might be capable of spreading to multiple organs through peripheral nerves, precipitating multiple organ failure independently of an underlying respiratory infection.

## Internalization of ACE2 as a Dominant Entry Mechanism

S-glycoproteins, expressed on the surface of SARS-CoV-2 virions, engage the ACE2 on host cells, and invade the cells either by membrane fusion or endocytosis. In order to initiate the membrane fusion, S-glycoproteins need to undergo cleaving by endogenous proteases, which enables them to engage the ACE2 more avidly (Ou et al., 2020). This feature is absent in other coronaviruses, including SARS-CoV-1 (Jaimes et al., 2020). Some proteases involved in this process have already been identified, e.g., furin and TMPRSS2 (Hoffmann et al., 2020b; Walls et al., 2020), however, other proteases might be also involved. Additionally, the docking of SRAS-CoV-2 to the cell membrane is facilitated by heparan sulfate proteoglycans on the host cell, which interact with S-glycoproteins (Mycroft-West et al., 2020). In SARS-CoV-2 S-glycoprotein, three novel glycosaminoglycan-binding motifs have been recently described, one of which is located at S1/S2 cleavage site (Kim et al., 2020). This finding further implies involvement of host cell surface proteoglycans in the process of cell entry.

When the proteases are unavailable, membrane fusion cannot happen, and binding of SARS-CoV-2 to ACE2 would result in endocytosis instead. Moreover, even when the proteases are available, the virions still prefer entry via endocytosis (Ou et al., 2020). Endocytotic entry in *Coronaviridae* dependents on the localization of their receptors in membrane lipid rafts, since lipid rafts mediate this process. This mechanism shares the same activating principles with renin-angiotensin-aldosterone system, suggesting their common phylogenic origin (Chen et al., 2012). The initiation of ACE2-dependent endocytosis in SARS-CoV-2 was reported to be dependent on phosphatidylinositole biphosphate (Ou et al., 2020). The protease-independent and lipid-raft-mediated entry might mimic physiological activation of the receptor by angiotensin II, which results in its recruitment to the intracellular renin-angiotensin system (Escobales et al., 2019; Abassi et al., 2020). We still do not know much of this system, nonetheless, its involvement in the various aspects of metabolic regulation of subcellular compartments is gradually being elucidated (Villar-Cheda et al., 2017; Shi et al., 2018; Sotomayor-Flores et al., 2020).

**FIGURE 1:**
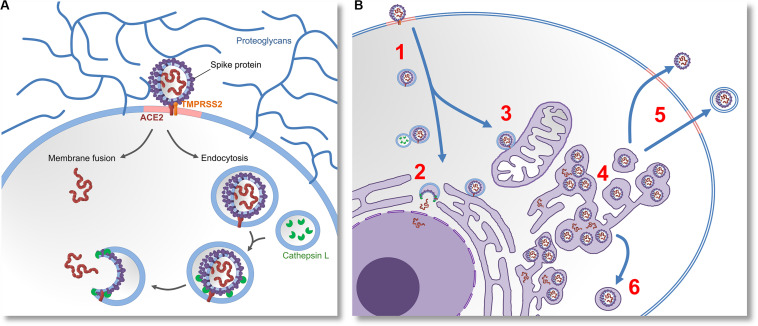
Molecular mechanisms of cell entry, replication and egression of SARS-CoV-2. **(A)** The virus invades the cell by docking on the cell-surface proteoglycans and engaging the ACE2 receptor with “Spike” (S) glycoprotein. If S glycoprotein is cleaved by host proteases (TMPRSS2), entry by membrane fusion or endocytosis would happen. If the cleavage does not occur, the virus would invade the cell via receptor-mediated endocytosis. Lysosomal proteases (Cathepsin L) eventually cleave the S glycoproteins. This enables them to induce membrane fusion and release the viral proteins and RNA into the cytoplasm. **(B)** After the virus enters the cell (1) it releases its genetic material (2), and replicates in the cell nucleus. Virions that do not get their S glycoproteins primed by lysosomal proteases would be further trafficked to various subcellular membrane compartments (3), which possibly modulates their metabolism and changes their morphology. This results in the emergence of a membranous system called reticulovesicular network. Translation of viral messenger RNA, synthesis of viral proteins and assembly of new virions takes place inside of this network and other intracellular vesicles (4). New virions leave the infected cell by budding through lipid rafts, either by membrane-fusion-mediated egression or by exosomes (5). Intracellular cleavage of S glycoprotein by furin or TMPRSS2 proteases would enable the virions to induce membrane fusion. Alternatively, assembly of virions inside of secretory vesicles would allow them to be transported to the apical cell membrane (6) or presynaptic membrane (in the case of neurons).

After ACE2 endocytosis, lysosomal cathepsin L proteases are normally trafficked to the endosome. It has been recently demonstrated that cathepsin L is capable of cleaving S-glycoproteins, enabling virions to initiate fusion, escape endosomes and release their proteins and genetic material into cytosol (Mao et al., 2020). By preferably relying on endocytosis instead of membrane fusion, SARS-CoV-2 likely postpones its detection by the immune system, because in this way fewer antigenic viral proteins are left on the cell surface (Marsh and Helenius, 2006). The mechanisms of cell entry are summarized in the Figure 1A.

## Exploitation of Subcellular Membrane Compartments and Intracellular Transport

An important feature of coronaviruses is that their replication-transcription complexes are associated with double membrane vesicles built from modified Golgi apparatus and endoplasmic reticulum (Snijder et al., 2020). The viruses extensively remodel the membranes of subcellular compartments into organelle-like and web-like structures, known as reticulovesicular networks (Knoops et al., 2008). A recent preprint electron microscopy study confirmed the same for SARS-CoV-2 (Belhaouari et al., 2020). The viral replication-transcription domains and assembled virions were also reported in autophagosomes (Prentice et al., 2004) and secretory vesicles (Krijnse-Locker et al., 1994; Salanueva et al., 1999; Verheije et al., 2008), implying that assembly and transport of new virions might take place during vesicular trafficking. For their replication and neuronal dissemination, neuroinvasive viruses must express proteins that control vesicular traffic (Enquist, 2012). Angiotensin II increases and mediates neuronal vesicular trafficking (Wang et al., 2001; Aschrafi et al., 2019), and since the receptor binding site of S-glycoproteins in SARS-CoV-2 is structurally similar to angiotensin II, the virus might be capable of increasing and modulating the neuronal vesicular trafficking system in the same manner (see Figure 1B). Moreover, as coronaviruses modify and assembly inside of structures derived from endoplasmic reticulum, we further suggest that SARS-CoV-2 could also utilize continuous longitudinally spanning endoplasmic reticula, which were described in the myelinated axons, and which are likely a continuation of the somatic organelles (Gonzalez and Couve, 2014). Since SARS-CoV-2 is a neurotropic virus, we suggest that, by binding to ACE2, it is able to disseminate via both vesicular transport and passive diffusion through axonal endoplasmic reticulum over large distances and at a fast pace.

New virions that are assembled in a reticulovesicular network are not immediately released out of the infected cell. Instead, they are accumulating in dedicated areas of its lumen (Knoops et al., 2008). Their egression is most likely elicited by fusion of the vesicles derived from the reticulovesicular network and plasma membrane in a process that seems to be dependent on interaction with lipid rafts (Chazal and Gerlier, 2003; Baglivo et al., 2020; Fantini et al., 2020) and autophagosomal proteins (Tanida et al., 2009). Since the surfaces of lipid rafts are much smaller than the viral envelopes, egression has to happen on sites where many lipid rafts cluster into a lipid microdomain (Lorizate and Kräusslich, 2011). This egression mechanism might be crucial for the induction of syncytia. S-glycoproteins of SARS-CoV-2 induce syncytia by transcellular transfections dependent on TMPRSS2 proteolytic activity (Ou et al., 2020). Apparently, the virion cannot directly induce a syncytium without proteases, likely because membrane fusion cannot be initiated. In such cases the budding would likely result in an endocytic transfection, enabling the virus to spread in a cell-to-cell fashion. SARS-CoV-2 was reported to show superior *in vitro* cell-cell fusion capacity compared to SARS-CoV-1 (Xia et al., 2020). Additionally, in some coronaviruses, soluble S-glycoproteins are secreted out of the infected cell, and are shown to induce syncytia independently of transfection (Masters, 2006).

Axonal dissemination by vesicular transport and passive diffusion, syncytium induction and cell-to-cell spread could explain the unexpectedly low viral load in the blood – possibly less than 1% of PCR blood tests in CoVID-19 patients yield a positive result (Wang et al., 2020b; Wölfel et al., 2020; Yu et al., 2020), suggesting that viremia likely does not underlie the multi-organ dissemination.

## Brain as a Hub for Further Dissemination

ACE2 is expressed in neurons of many brain regions (Doobay et al., 2007). It can bind to integrins and modulate their signaling (Clarke et al., 2012). Integrins are transmembrane receptors responsible for signal transduction between a cell and extracellular matrix, and are abundantly expressed in synapses and terminals of sensory neurons that mediate pain (Dina et al., 2004), implying a possible colocalization with ACE2 at those sites. Furthermore, an integrin-binding motif in S-glycoprotein of SARS-CoV-2 was recently identified, suggesting that they might be alternative receptors for the virus, and an ACE2-independent infection in integrin-expressing cells might be possible (Sigrist et al., 2020). The presence of SARS-CoV-2 in the cerebrospinal fluid was recently reported in a case of viral encephalitis (Moriguchi et al., 2020), and the virus was directly observed in the brain cells of deceased CoVID-19 patients (Paniz-Mondolfi et al., 2020), confirming its neurotropic nature. Based on these findings and recent reports (Cheema et al., 2020; Colavita et al., 2020), we propose that nerve terminals in the oral and nasal mucosa, conjunctiva and eyes, as well as the olfactory nerves, might be potential entry sites for SARS-CoV-2 neurotropic infections. *Post-mortem* MRI findings revealed asymmetric olfactory bulbs in four deceased CoVID-19 patients, further implying that olfactory neuroepithelium might be an entry point for the virus (Coolen et al., 2020).

**FIGURE 2:**
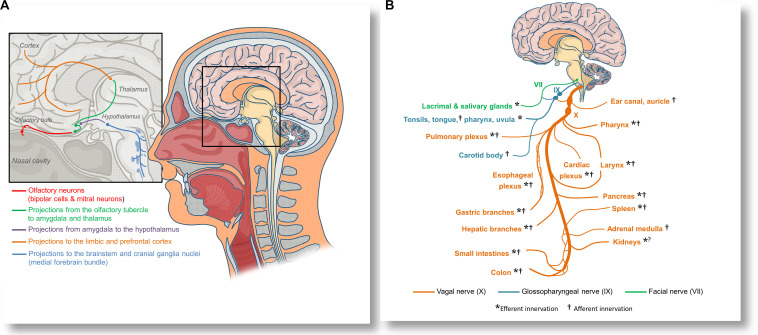
Anatomical overview of the proposed olfactory inoculation and axonal dissemination pathways of SARS-CoV-2. **(A)** Olfactory neurons are only a synapse away from the central nervous system. SARS-CoV-2 has been reported to infect olfactory neuroepithelium and to invade the olfactory bulbs via cribriform plate. By exploiting the anterograde axonal transport in the olfactory tract, the virus could infect neurons of the olfactory tubercle and spread to the amygdala and thalamus, from where it might further invade the cingular and orbitofrontal cortex. By exploiting the axonal transport in fibers projecting into the hypothalamus, the virus may infect cranial ganglia nuceli via the medial forebrain bundle. **(B)** SARS-CoV-2 could also disseminate to various organs and tissues by axonal transport in the vagal nerve (X). Immediately after leaving the skull, the vagus establishes anastomoses (connections) with the glossopharyngeal nerve (IX), allowing the virus to spread to the oropharyngeal mucosa, or alternatively, to use the same route for neuroinvasion. Glossopharyngeal fibers that cross to the facial nerve (VII) could be an additional pathway for dissemination or neuroinvasion. The vagal nerve innervates many tissues and organs that can be affected in CoVID-19, including the pharynx, larynx, lungs, the heart, esophagus, stomach, liver, gallbladder, pancreas, spleen, adrenal medulla, kidneys, muscles, and glands of a part of the intestines, as well as lymphatic tissue in the correspondent intestinal mucosa. By disrupting the vagal innervation, SARS-CoV-2 could also impair the activity of the cholinergic inflammatory reflex, and precipitate dysregulated immune responses in many organs.

CoVID-19 patients frequently present with hyposmia and dysguesia (Bagheri et al., 2020; Lechien et al., 2020), and both ACE2 and TMPRSS2 proteases are expressed in olfactory neuroepithelium (Fodoulian et al., 2020). Moreover, Dubé et al. (2018) directly observed propagation of a human coronavirus to the brainstems of mice following the intranasal and intralingual inoculations, suggesting that SARS-CoV-2 might be able to spread to the brainstem either directly via olfactory nerves, or alternatively, through orofacial nerve fibers via cranial ganglia. A non-peer-reviewed report demonstrated the presence of SARS-CoV-2 in the trigeminal ganglia, olfactory epithelium, olfactory bulbs, brainstem, uvula, conjunctiva and cornea in some deceased patients (Meinhardt et al., 2020). Olfactory inoculation likely involves propagation to the piriform cortex and amygdala, and further spreading through the medial forebrain bundle to the brainstem (see Figure 2A). Lateral fiber stream of the medial forebrain bundle projects caudally to the solitary tract and dorsal vagal nuclei (Holstege, 1987). Replication of the virus in the solitary tract neurons may also explain the reported dysgeusia. Spreading through the orofacial sensory fibers would be especially convenient for the virus, since their pseudounipolar somata, which reside in the cranial ganglia, could be plausible persistent infection sites or intermediary replication posts. This could facilitate either further brainstem invasion by axonal transport or allow for an exocytosis-endocytosis-mediated transfection of other fibers passing through the ganglia. Such virion-containing endocytes could establish membrane contact sites with axonal endoplasmic reticulum (Eden, 2016), enabling the virion to freely diffuse along the axon using the organelle lumen. Passive diffusion of coronavirions in axons was reported both *in vitro* and *in vivo* (Dubé et al., 2018). However, it is possible that vesicular transport might prevail *in vivo*. Although hematologic dissemination to the brain cannot be excluded, the observed discrepancy between a significant incidence of neurological manifestations (Mao et al., 2020) and a low yield of positive blood tests (Wang et al., 2020b; Wölfel et al., 2020; Yu et al., 2020) suggests that viremia is unlikely to be a major contributor to the brain infection.

**FIGURE 3:**
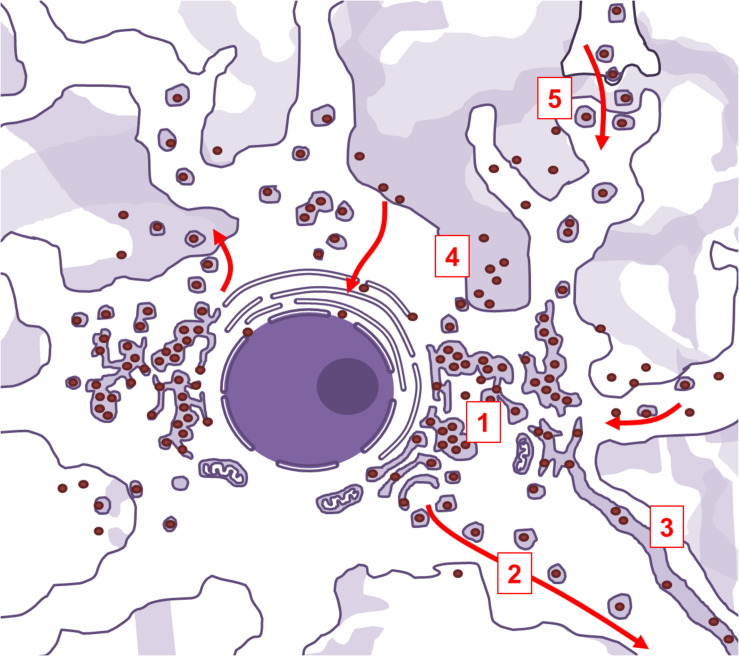
Aspects of intracellular vesicular trafficking that may be exploited by SARS-CoV-2 in infected neurons. Neurons are polarized cells with abundance of intracellular endocytic pathways. Life cycle of SARS-CoV-2 is compatible with the possibility of exploiting those pathways. The virus extensively modifies subcellular organelles into a reticulovesicular network, a structure where viral membrane-bound replication-transcription complexes are situated and where new virions are being assembled (1). This elaborate network is connected to secretory Golgi compartments, allowing the newly assembled virions to be trafficked to the synapse via kinesin-mediated anterograde axonal transport (2). The reticulovesicular network is also continuous with endoplasmic reticulum. In myelinated projection neurons, endoplasmic reticulum extends along the axon, which might enable the virions to freely diffuse inside its lumen (3). Newly assembled virions can also directly leave the infected neurons by membrane-fusion or by exosomes (4), and infect the nearby cells. Trans-synaptic spreading is confirmed in some beta-coronaviruses, and could be possible in SARS-CoV-2 as well (5).

Viral penetration into the central nervous system through peripheral fibers is a multi-step process. In order to reach neuronal soma from the periphery, the virus needs to exploit the retrograde axonal transport machinery. SARS-CoV-2 uses ACE2-mediated endocytotic pathway for internalization and intracellular transport, and in the case of SARS-CoV-1 infection, endosomes containing virion/ACE2 complexes are trafficked to the perinuclear area (Wang et al., 2008). The virus might use this intrinsic clathrin-independent intracellular ACE2 endocytic transport to reach the perikaryon. However, for a successful further invasion, it would also need to be able to cross synaptic membranes. Another beta-coronavirus was shown to be capable of trans-synaptic propagation by presynaptic exocytosis and postsynaptic endocytosis (Li et al., 2013), which suggests that SARS-CoV-2 could use the same mechanism. Anterograde axonal transport is mediated by kinesin molecular motors, and allows for trafficking of vesicles and organelles from the soma to the axon and synaptic terminals (Berth et al., 2009). Since the virus replicates and assembles inside of vesicles derived from the endoplasmic reticulum and Golgi apparatus, it could also exploit the already present kinesin-mediated anterograde transport to propagate further along the axons. Lateral transfections, i.e., cell-to-cell or axo-axonal spreading via exocytosis, could be also possible. Exosomal pathways are hypothesized to contribute to viral dissemination (Khan et al., 2017), and it was demonstrated that ACE2 trafficking could involve exosome-mediated cell-to-cell transfer (Wang et al., 2020a). Arguably, this mechanism could allow the infection to spread from neurons to cerebrovascular endothelial cells, and *vice versa*. The ways the virus might exploit intracellular vesicular trafficking in neurons are summarized in Figure 3.

Once the virus has reached the brainstem and spinal cord, it could access practically every organ system in the body. By infecting the vagal nuclei alone, the virus may be capable of dissemination to the lungs, heart, liver, intestines and kidneys, as well as of impairing the vagal activity (see Figure 2B). This might precipitate multiple organ injury independently of an underlying respiratory pathology. In a murine model of neuronal infection with human coronavirus OC43, the viral RNA was detected in the livers of three out of nine animals in spite of undetectable viral loads in the blood (Dubé et al., 2018). This finding supports the possibility of dissemination through vagal fibers. Additionally, viral shedding at the periphery could also be associated with activation of the integrin signalization on peripheral nerve terminals, which could enable the virus to attenuate local algesic and inflammatory response, hindering the immune reaction to its shedding (Dina et al., 2004; Moon et al., 2009; Hu et al., 2016). Alternatively, the virus may establish a persistent neuronal infection, and stay dormant for a certain period until eventual reactivation. This is a common strategy in some neuroinvasive viruses (Koyuncu et al., 2013).

Other manifestations that are considered atypical for a respiratory infection, such as coagulopathy (Iba et al., 2020), thrombosis (Helms et al., 2020; Leisman et al., 2020), vasculitis (Castelnovo et al., 2020; Sachdeva et al., 2020; Verdoni et al., 2020) and dysregulated inflammatory responses (Blanco-Melo et al., 2020; Leisman et al., 2020) were also reported in CoVID-19 patients. Previous studies showed that vagal activity is an important factor in anti-inflammatory modulation and inhibition of prothrombotic events in the innervated tissues (de Jonge et al., 2005; Westerloo et al., 2006; Koopman et al., 2016; Li et al., 2016; Cacho et al., 2020), and SARS-CoV-2 could be capable of hijacking axonal transport in the vagal nerves, impairing their signaling in the cholinergic anti-inflammatory pathway (Johnston and Webster, 2009; Pavlov and Tracey, 2012). We propose that vagal dysfunction might significantly contribute to exaggerated immune responses and thromboembolic incidents in some CoVID-19 patients. Interestingly, vagal neuropathies due to upper-respiratory viral infections are already clinically recognized as contributors to various para-infectious and post-infectious sequelae (Amin and Koufman, 2001; Rees et al., 2009; Chung et al., 2013; Niimi and Chung, 2015).

Due to the dynamics of the active transport and passive diffusion in axons, the brain infection might develop weeks after the virus exposure or development of primary respiratory infection, giving rise to the possibility that patients with severe clinical presentation and multiple organ affection might have contracted the virus earlier than assumed. The exact time needed for the virus to invade the brain in humans is unknown, and it certainly depends on the entry route and inocculation dose. In mice, a strain of human coronavirus was detected in the olfactory bulbs as early as 2 days after intranasal inoculation, in the cortex and brainstem 3 days after inoculation, and in the spinal cord 5 days after inoculation (Dubé et al., 2018).

## Implications and Perspectives

Based on the presented concept, we would like to suggest that respiratory and neuronal types of CoVID-19 may be distinct clinical entities. These two types might present independently, as a respiratory infection without brain infection and *vice versa*, concomitantly or consecutively. Due to different entry routes, the two types would likely have different incubation periods and different occurrence rates of initial symptoms, which could explain the observed variability in both parameters (Day, 2020; Jiang et al., 2020; Wan et al., 2020). However, wide dispersion of reported values could be also due to limited sample sizes in the initial reports.

Increased susceptibility to a particular type of the disease might be driven by underlying conditions. ACE2 expression is increased in patients with morbidities associated with metabolic syndrome (Pinto et al., 2020), and those patients are also more likely to develop neurological manifestations (Mao et al., 2020). Patients with such conditions who develop CoVID-19 respiratory infections might be at risk of more serious CoVID-19 neuronal infections which could in turn result in the virus dissemination to multiple organs through peripheral nerves. Most patients, however, do not develop brain infection. It is important to note that the nasal mucosa possess mechanisms that efficiently prevent neuroinvasion via olfactory nerves, such as nasal secretion, mucus barrier formation, pathogen recognition receptors (Kalinke et al., 2011) and cyclic shedding and replacement of olfactory neurons with the new ones (Loseva et al., 2009). Another host protective response was reported to be apoptosis of olfactory neurons (Mori et al., 2004). Conditions that interfere with these mechanisms might compromise their protective roles against neuroinvasive infectious agents. Aging, diabetes, and hypertension are associated with less efficient nasal mucocilliary clearance (Selimoglu et al., 1999; Yue, 2007; Proença de Oliveira-Maul et al., 2013), and aging might also precipitate reduced olfactory nerve replenishment (Enwere et al., 2004; Brann and Firestein, 2014). This would additionally explain the observed higher incidence of neurological involvement in patients with these comorbidities. For most otherwise healthy and younger individuals, respiratory epithelium would be the primary and likely the only site of infection, whereas the aforementioned high-risk groups might be more susceptible to both neuronal and respiratory types of CoVID-19.

Theoretically, a primary lung infection could also progress to a brain or spinal cord infection via retrograde axonal transport through peripheral nerves. However, more aggressive immune responses to viral pathogens in peripheral tissues compared to the ones in the central nervous system would likely impede such a scenario. Due to irreplaceability of neurons, the immune reactions to viral infection in the brain do not include cytolytic responses, and are therefore less efficient in containing and clearing intracellular pathogens (Griffin, 2003). Olfactory neurons, although replaceable, are in an immediate proximity to the central nervous system, which makes them an anatomically and immunologically more plausible route for successful neuroinvasion. Nevertheless, a primary lung infection in some patients could still progress to acute respiratory distress syndrome without or independently of neuronal infection. Such lung injuries might be due to suboptimal host reaction to the infection, possibly characterized by a weak antiviral response and elevated expression of proinflammatory cytokines, as demonstrated by an *in vitro* study (Blanco-Melo et al., 2020). Still, many CoVID-19 patients who develop respiratory infection without neural involvement could have better clinical outcomes, whereas a combination of direct cytopathic effects, vagal neuropathy and centrally driven lung injuries could be associated with less favorable outcomes.

We propose that the original type of cell in which the virion assembly and budding took place could be identified based on the lipid profile of the viral particles. The lipid composition of retroviral envelopes corresponds to the lipid profile of the membrane lipid rafts at which the budding took place (Ono, 2010; Waheed and Freed, 2010). Since lipid rafts of the brain have a distinctive lipid profile rich in specific gangliosides (Vajn et al., 2013; Schnaar et al., 2014), by comparing it to the lipid profile of the virions, it could be possible to confirm the neuronal origin of SARS-CoV-2 in peripheral tissues.

A proportion of purportedly asymptomatic or oligosymptomatic carriers could suffer a less severe CoVID-19 neuronal infection, with subtle neuropsychiatric manifestations without respiratory involvement. RNA viruses are known to be able to persistently infiltrate CNS as well as to cause subacute psychiatric and neurological symptoms and post-infectious sequelae, such as cognitive impairment, seizures, ataxia, psychiatric illnesses, chronic fatigue syndrome, etc. (Klein et al., 2019; Bo et al., 2020). To the best of our knowledge, so far reported neurological manifestations in CoVID-19 patients include hyposmia, dysgeusia (Lechien et al., 2020), convulsions (Karimi et al., 2020), neurogenic syncope (Canetta et al., 2020), meningoencephalitis (Moriguchi et al., 2020), Guillain-Barré syndrome (Zhao et al., 2020b), intracerebral hemorrhage (Sharifi-Razavi et al., 2020), acute hemorrhagic necrotizing encephalopathy (Poyiadji et al., 2020), acute post-infectious myelitis (Zhao et al., 2020c), cerebrovascular diseases (Mao et al., 2020), vertigo, nausea, headaches (Mao et al., 2020; Nie et al., 2020), demyelination (Zanin et al., 2020), and cortical blindness (Kaya et al., 2020), but the causative or coincidental nature of these findings is yet to be determined. It is important to point out that some of the reported neurological symptoms could also be caused by hypoxia as a consequence of lung injury. However, not all CoVID-19 patients who developed neurological symptoms suffered pulmonary insufficiency, and the presence of subtle neuropsychiatric abnormalities in the subclinical cases might be actually underreported (Zhang et al., 2020b).

The fetus seems to be protected from the axonal invasion of SARS-CoV-2 from the infected mother by factors that inhibit nerve growth on the maternal side of the umbilicus and placenta (Marzioni et al., 2004). Both amniotic fluid and umbilical cord blood samples were reported to test negative to SARS-CoV-2, and no vertical transmission was reported (Chen et al., 2020a), except for a recent report of three cases of neonatal CoVID-19, in which vertical transmission could not be ruled out (Zeng et al., 2020). Since ACE2 is expressed in the uterus and placenta (Valdes et al., 2013), a possibility of viral interference with expression of the factors that mediate nerve growth inhibition must not be dismissed. In addition, CoVID-19-related thromboembolic placental injuries were recently described (Baergen and Heller, 2020).

Development of neuronal CoVID-19 infection might explain a growing number of positive PCR tests in recovered patients even weeks after hospital discharge (Lan et al., 2020; Xing et al., 2020; Zhang et al., 2020c). Viral shedding at nerve terminals of pulmonary epithelium and nasopharyngeal mucosa could explain the sustained presence of SARS-CoV-2 in throat and nasal swabs, implying that a carrier state could persist over a significant timespan. Although prolonged positivity could theoretically be explained by presence of remnants of unviable viral RNA, we believe this is an unlikely explanation. Physiological nasopharyngeal washing and, possibly, activity of certain canonical ribonucleases in the respiratory mucosa (Koczera et al., 2016) would not allow for a sustained presence of the viral RNA weeks after recovery. By analogy, viral shedding may be also possible on the enteric nerve terminals, maintaining the detectability of the virus in enterocytes and stool even after apparent recovery. Hu et al. (2020) have recently reported that SARS-CoV-2 can persist in stool samples longer than in the respiratory tract in recovered patients who were previously without gastrointestinal symptoms.

The damage to multiple organs in some patients may as well be explained by hypoxia and cytokine storm (Bonow et al., 2020; Mehta et al., 2020; Pei et al., 2020; Yang et al., 2020). Even so, hypoxia and cytokine storm do not accompany all cases of organ damage (Kochi et al., 2020; Zhang et al., 2020a), and the correlation of incidence of hypercytokinemia and presence of viral RNA in blood (Chen et al., 2020b), in spite of practically non-existent viremia, suggests that cytokine storm might be preceded and driven by organ damage and subsequent release of viral antigens from necrotic cells. As a matter of fact, the virus presence was confirmed in vascular endothelial cells (Varga et al., 2020) and multiple organs in deceased patients (Puelles et al., 2020), and different mechanisms of organ failure do not necessarily exclude each other. Detrimental pro-thrombotic and pro-inflammatory state could also be driven by hypothesized SARS-CoV-2-induced vagal neuropathy (Li et al., 2011; Huston, 2012), and eventual development of neurogenic pulmonary edema secondary to an infection-related cerebrovascular event might contribute to the ultimate cardiopulmonary failure (Davison et al., 2012).

It was also suggested that possible fecal-oral transmission may explain the gastrointestinal symptoms in CoVID-19 (Cha et al., 2020; Steardo et al., 2020; Tian et al., 2020), even though SARS-CoV-2 is not stable in the media with pH <3 (Chin et al., 2020). Nonetheless, SARS-CoV-2 was still detected in stool and gastrointestinal mucosa (Xiao et al., 2020), but stool tested positive even in patients who did not have gastrointestinal symptoms (Zhang et al., 2020c). Still, the proposed fecal-oral route does not exclude the possibility of axonal dissemination of SARS-CoV-2 to gastrointestinal tract via vagal fibers and spinal nerves. Another possibility might be an infection of the gallbladder or biliary ducts (Zhao et al., 2020a), in which case the virus in stool would be of biliary origin.

Finally, pharmacologic approaches that would hinder the exploitation of the neuronal endocytic trafficking by SARS-CoV-2 could be an effective treatment for the infection. Chloroquine and its derivatives disrupt endocytosis and vesicular trafficking by endosomal alkalization and inhibition of autophagy, also interfering with terminal glycosylation in ACE2, which hinders its interaction with S-glycoproteins (Liu et al., 2020). These medications are already being clinically used in CoVID-19 patients. Other autophagy inhibitors, such as azithromycin are also commonly used (Gautret et al., 2020). Therefore, we suggest that the treatment of CoVID-19, due to its neuroinvasive properties, should focus on interfering with viral hijacking of the cellular endocytic trafficking system and axonal transport. A study of rat primary superior cervical ganglia culture revealed that emetine (translation elongation inhibitor) may be used as inhibitory modulator of rabies virus axonal transport (MacGibeny et al., 2018), implying a possible therapeutic approach for SARS-CoV-2. In the case of poliomyelitis virus infection in rats, vinblastine (inhibitor of tubulin polymerization) was shown to hinder retrograde axonal transport of the virus when applied topically to infected peripheral nerves (Ohka et al., 2004). Microtubule-associated inhibitors, such as vinblastine, vincristine, paclitaxel, colchicine, nocodazole and other inhibitors of retrograde axonal transport, such as macrolide drug mycalolide B (Cavolo et al., 2015), could be used to investigate the mechanisms underlying retrograde axonal transport of SARS-CoV-2 *in vivo*. However, these drugs do not alter the redistribution and abundance of viral proteins, and do not influence the viral replication (Wu et al., 2019). Moreover, treatment with these agents was reported to induce reactivation of varicella-zoster virus infection along with their neurotoxic effects. HSP90 inhibitor geldanamycin is suggested as a potential drug in the treatment of CoVID-19 (Sultan et al., 2020), and SARS-CoV-2 proteases inhibitor quercetine is being studied as a prophylaxis and treatment option (Onal and Semerci, 2020). It also affects the cytoskeletal signaling by inhibiting protein kinase C. Another potential treatment option for CoVID-19 are rho-kinase inhibitors, such as fasudil, ripasudil, and netarsudil (Abedi et al., 2020; Calò et al., 2020). Interestingly, all these compounds share a quinoline backbone moiety. Additionally, since neurotropic viruses have to propagate across the synapses, neutralizing antibodies could be used to stop them from spreading from neuron to neuron, as it was demonstrated in animal models of West Nile virus neuronal infection (Oliphant et al., 2005; Samuel et al., 2007). Another group of potential axonal transport modulators could be bioactive compounds isolated from marine organisms. Some of them are reported to inhibit molecular motors underlying anterograde or retrograde axonal transport (kinesin and dynein, respectively), and several compounds are proposed to interfere with autophagosomal pathways in neurons (White et al., 2016).

The model we put forward clarifies a wide range of clinically observed phenomena in CoVID-19 patients (see Supplementary Table S1). Detection of viral particles in peripheral nerves, together with recent findings of brainstem and cranial ganglia infection, as well as other findings summarized in this paper, could confirm the axonal dissemination of SARS-CoV-2. If correct, this would significantly affect our understanding of this novel disease and its potential long-term sequelae. This would warrant modifications in many aspects of diagnostics, treatment and follow-up of CoVID-19 patients. The proposed model could also be utilized by many other viruses – chronic persistence in the host’s nervous system and eventual reactivations with shedding in the respiratory or gastrointestinal mucosa could be an effective survival and spreading strategy for a virus. Finally, the presence of antibodies to other coronaviruses in the cerebrospinal fluid of patients with Parkinson’s disease and some psychiatric disorders (Fazzini et al., 1992; Severance et al., 2009; Okusaga et al., 2011) points to the possibility that these and similar pathologies might be triggered by viral infections. Vagal atrophy observed in patients with Parkinson’s disease (Walter et al., 2018), might also be secondary to bulbar lesions caused by a coronavirus infection. The proposed model of axonal dissemination and vagal dysfunction could give us novel insights not only into CoVID-19, but also into hypothesized common viral etiology of certain neurodegenerative and psychiatric disorders and their systemic manifestations. Therefore, we believe this idea merits further investigation.

As a closing remark, it is important to add that most individuals diagnosed with CoVID-19 will likely convalesce without developing neuronal infection. Moreover, the sole presence of proviral genomes in the brain does not imply a definite corresponding clinical correlate. Many viruses have already left their genetic imprints in our DNA, and thus became a part of our evolutionary heritage, and a part of us.

## Data Availability Statement

All datasets generated for this study are included in the article/Supplementary Material.

## Author Contributions

MF, SM, and MH devised the main conceptual ideas. MF drafted the initial manuscript. MF, SM, MB, ST, MZ, AR, DM, ZD, and MH reviewed and revised the manuscript, and expanded the original concept. All authors contributed to the article and approved the submitted version.

## Conflict of Interest

The authors declare that the research was conducted in the absence of any commercial or financial relationships that could be construed as a potential conflict of interest.
