# Supplementary material for: SARS-CoV-2 Dissemination Through Peripheral Nerves Explains Multiple Organ Injury
Source: Front Cell Neurosci. 2020 Aug 5;14:229. doi: 10.3389/fncel.2020.00229 (PMC7419602; doi:10.3389/fncel.2020.00229)
Supplement: Supplementary file 1 [file Table_1.docx]

# Supplementary material

**Table S1.** Clinically documented symptoms and manifestations of CoVID-19 and proposed pathophysiological mechanism(s).

| *Clinically observed phenomena* | *Proposed mechanism(s)* | |  |
| --- | --- | --- | --- |
| **Neurological manifestations** |  | |  |
| Dysgeusia (Lechien et al., 2020) | Invasion of gustatory receptors, viral replication in the solitary tract neurons, changes in peripheral and central circuitry involved in taste perception. | |  |
| Hyposmia (Lechien et al., 2020) | Invasion of olfactory neuroepithelium, shedding of infected olfactory  receptors and/or olfactory bulb interneurons as a protective host response, viral propagation and infection of the piriform cortex. | |  |
| Convulsions (Karimi et al., 2020) | Damage to the inhibitory interneurons due to neuronal infection, hypoxic damage, metabolic disturbances. | |  |
| Meningoencephalitis (Moriguchi et al., 2020) | Brain and meningeal infection. | |  |
| Guillain–Barré syndrome (Zhao H. et al. 2020) | Damage to pseudounipolar neurons in the spinal ganglia, post-infectious etiology | |  |
| Intracerebral hemorrhages, acute hemorrhagic necrotizing encephalopathy (Sharifi-Razavi et al., 2020; Poyiadji et al., 2020) | Damage to the vascular endothelium due to viral infection, disbalance in neuro-glial coagulonome. | |  |
|  |  |  |  |
| Ischemic stroke (Mao et al., 2020) | Hypoxia, thromboembolism as a consequence of vascular endothelium infection and/or vagal neuropathy. | |  |
| Acute (post-infectious) myelitis (Zhao K. et al., 2020) | Infection of spinal cord neurons and infection-related inflammation. | |  |
| Vertigo, nausea, headaches (Mao et al., 2020; Nie et al., 2020) | Hypoxia, osmotic disbalance due to hypoenergosis, viral invasion of the vestibular nuclei and cerebellar projections, viral propagation through axons of vestibular nerves. | |  |
| Demyelination (Zanin et al., 2020) | Metabolic changes in axons that affect axo-myelin stability and survival of oligodendrocytes due to axonal dissemination and/or infection-related neuronal loss, hypoxic leukoencephalopathy | |  |
| Cortical blindness (Kaya et al., 2020) | Hypoxia, neuronal viral infection, thromboembolic incident | |  |
| Posttraumatic stress symptoms (Bo et al., 2020) | Viral invasion of amygdala or neurons projecting to amygdala, reactive etiology due to psychosocial circumstances | |  |
| Depression (Zhang J. et al., 2020b) | | Monoaminergic hypoactivity due to cytokine-mediated trophic and metabolic changes in neurons, viral infection of the monoaminergic neurons and circuitry involved in stress response (amygdala, hypothalamus), reactive etiology due to psychosocial circumstances |  |
| **General manifestations of infection** |  | |  |
| Fever (Wan et al., 2020; Lai et al., 2020) | Effect of pyrogenic cytokines. | |  |
| Cough (Wan et al., 2020; Lai et al., 2020) | Protective response to respiratory epithelium infection, viral effects on the vagal nerve. | |  |
| Fatigue (Wan et al., 2020; Lai et al., 2020) | Investment of energy into immune response, hypoxia, monoaminergic hypoactivity due to inflammation or neuronal infection (including persistent viral infection). | |  |
| Myalgia (Wan et al., 2020; Lai et al., 2020) | Visceral pain caused by anaerobic metabolism in muscles due to infection-related energy metabolism shifts, hypoxia, viral affection of sensory nerves. | |  |
| **Respiratory manifestations** |  | |  |
| Pharyngalgia (Wan et al., 2020; Lai et al., 2020) | Continuous irritation of mucosa by local inflammatory response, virally induced neuropathy. | |  |
| Sputum production (Wan et al., 2020; Lai et al., 2020) | Viral infection of respiratory epithelium. | |  |
| Rhinorrhoea (Wan et al., 2020; Lai et al., 2020) | Enhanced mucosal washing as a protective host response, viral effect on innervation of serous and mucous glands of the nose and nasopharynx (e.g. facial nerve infection, viral replication in the pterygopalatine ganglion). | |  |
| Dyspnea (Wan et al., 2020; Lai et al., 2020) | Hypoxia, direct viral lung injury, cytokine-response-related lung injury, neurogenic edema, viral effects on the vagal nerve (autonomic dys-innervation). | |  |
| Pneumonia (Hani et al., 2020) | Viral infection of alveolar/bronchial epithelia. | |  |
| Hemoptysis (Wan et al., 2020) | Expulsion of blood and detritus from the injured lung tissue. | |  |
| Acute Respiratory Distress Syndrome (Pan et al., 2020) | Loss of functional respiratory tissue due to direct cytopathic effects of the virus, exaggerated local proinflammatory response, virally induced vagal denervation/neuropathy, neurogenic edema. | |  |
| **Gastrointestinal and hepatic manifestations** |  | |  |
| Diarrhea (Cha et al., 2020; Tian et al., 2020) | Infection of intestinal mucosa, disruption of enteric innervation. | |  |
| Emesis (Cha et al., 2020; Tian et al., 2020) | Hypoxia, viral effects on the brainstem (area postrema), gastroparesis due to virally induced vagal injury. | |  |
| Abdominal pain (Cha et al., 2020; Tian et al., 2020) | Hypoxia, sensory fiber sensitization caused by a local viral inflammation, virally induced vagal injury, axonal transport of the virus through sensory fibers. | |  |
| Anorexia (Cha et al., 2020; Tian et al., 2020) | Viral hypothalamic invasion. | |  |
| Gastro-intestinal bleeding (Cha et al., 2020; Tian et al., 2020) | Hypoxic injury to the gastro-intestinal tract, vascular endothelium viral infection. | |  |
| Abnormal liver enzymes (Cha et al., 2020) | Viral hepatic infection | |  |
| **Ophthalmic manifestations** |  | |  |
| Conjuctival hyperemia (Colavita et al., 2020) | Conjuctival inflammation due to viral infection. | |  |
| Chemosis (Colavita et al., 2020) | Angioedema caused by viral infection of an eye. | |  |
| Epiphora (Colavita et al., 2020) | Reflex response to viral conjunctivitis, viral affection of innervation to the lacrimal gland (facial nerve or ophthalmic branch of the trigeminal nerve). | |  |
| Keratoconjuctivitis (Cheema et al., 2020) | Conjunctival and corneal inflammation due to viral infection. | |  |
| **Miscellaneous** |  | |  |
| Thrombosis, coagulopathy (Iba et al., 2020) | Disseminated intravascular coagulation due to cytokine storm, thrombosis due to endothelial viral infection, pro-thrombotic state due to disruption of parasympathetic innervation (e.g. vagal neuropathy). | |  |
| Cytokine storm (Yang et al., 2020) | Hyperproduction of cytokines amidst virus-induced cell/tissue injury, viraemia, exaggerated immune response due to viral effects on vagal anti-inflammatory innervation. | |  |
| Cutaneous manifestations (Sachdeva et al., 2020) | Viral migration via skin innervating axons, vasculitis-related cutaneous manifestations due to viral endothelitis and/or immune reaction. | |  |
| Baroreflex dysfunction (Canetta et al., 2020) | Viral infection of the solitary tract neurons, virally induced neuropathy of carotid sinus branch of glossopharyngeal nerve, virally induced ACE2 internalization in the solitary tract neurons | |  |
| Kidney injury (Pei et al., 2020) | Kidney infection (e.g. through renal branches of the vagal nerve, other nerve fibers and/or hematologic route), thromboembolic injury. | |  |
| Myocardial injury and arrhythmias (Bonow et al., 2020; Kochi et al., 2020) | Coronary thromboembolic incidents (see Thrombosis entry), parasympathetic denervation due to virally induced vagal injury, virus-mediated ACE2 internalization, hypoxia, direct infection of myocites. | |  |
| Placental injury (Baergen et al., 2020) | Thromboembolic injury (see Thrombosis entry). | |  |
